# The Role of Platelets in Hypoglycemia-Induced Cardiovascular Disease: A Review of the Literature

**DOI:** 10.3390/biom13020241

**Published:** 2023-01-27

**Authors:** Ahmed Ali Gebril Ali, Sara Anjum Niinuma, Abu Saleh Md Moin, Stephen L. Atkin, Alexandra E. Butler

**Affiliations:** 1 School of Medicine, Royal College of Surgeons in Ireland and Medical University of Bahrain, Busaiteen 15503, Bahrain; 2 Research Department, Royal College of Surgeons in Ireland and Medical University of Bahrain, Busaiteen 15503, Bahrain

**Keywords:** platelets, type 2 diabetes, cardiovascular disease, cardiovascular risk, therapeutics

## Abstract

Cardiovascular diseases (CVDs) are the leading cause of death globally as well as the leading cause of mortality and morbidity in type 2 diabetes (T2D) patients. Results from large interventional studies have suggested hyperglycemia and poor glycemic control to be largely responsible for the development of CVDs. However, the association between hypoglycemia and cardiovascular events is also a key pathophysiological factor in the development of CVDs. Hypoglycemia is especially prevalent in T2D patients treated with oral sulfonylurea agents or exogenous insulin, increasing the susceptibility of this population to cardiovascular events. The adverse cardiovascular risk of hypoglycemia can persist even after the blood glucose levels have been normalized. Hypoglycemia may lead to vascular disease through mechanisms such as enhanced coagulation, oxidative stress, vascular inflammation, endothelial dysfunction, and platelet activation. In the following review, we summarize the evidence for the role of hypoglycemia in platelet activation and the subsequent effects this may have on the development of CVD. In addition, we review current evidence for the effectiveness of therapies in reducing the risk of CVDs.

## 1. Introduction

For optimal metabolic function, an adequate supply of glucose is required. Potential complications, such as impaired target organ function, may arise if glucose levels fall and hypoglycemia ensues [1]. The American Diabetes Association has classified hypoglycemia into three levels [2]. The first level is for blood glucose levels between 70 mg/dL and 54 mg/dL; the second level for blood glucose levels less than 54 mg/dL; and the third level for severe hypoglycemia, indicated by “altered mental and/or physical status requiring assistance for treatment of hypoglycemia” [2]. Hypoglycemia can also be asymptomatic, detectable only by measurement of plasma glucose [3]. Common symptoms of hypoglycemia are fatigue, weakness, hunger, and tremulousness. A blood glucose level less than 70 mg/dL is considered as clinically significant regardless of the severity of symptoms [2].

Hypoglycemia is especially prevalent in patients with type 1 diabetes (T1D) treated with exogenous insulin, or T2D being treated with oral sulfonylurea agents or exogenous insulin [4,5]. Studies indicate that the incidence of hypoglycemia among the insulin-treated T2D population is about one-third of that seen in patients with T1D but the greater number of patients with T2D means that a greater number of these patients may suffer a hypoglycemic event, especially in patients with a longer duration of diabetes [6]. The occurrence of hypoglycemia in non-diabetic patients is rare and has been estimated to be about 13 episodes per 10,000 admissions in one year [7]. However, studies have indicated that non-diabetic patients diagnosed with hypoglycemia are more likely to die than diabetic patients due to the underlying pathology [7,8]. Hypoglycemia has been linked to an increase in cardiovascular risk and mortality as seen in the ACCORD study where intensive therapy to optimize HbA1c led to an increased mortality rate [9]. The pathophysiological effects of hypoglycemia include activation of the autonomic nervous system, an increase in counterregulatory hormones, and consequently an increase in myocardial workload and oxygen uptake [10]. Moreover, hypoglycemia triggers proinflammatory cytokines, impaired fibrinolysis, coagulation, and endothelial dysfunction, which can lead to the development of CVDs [10,11,12], with the increased activation and aggregability of platelets being a significant contributing factor.

Platelets have been termed the “nexus of vascular diseases” due to their impact on CVDs [13]. The binding of platelets to the disrupted endothelium can lead to platelet activation, an effect further enhanced by circulating catecholamines [14,15]. Platelets function as the first line of defense against injuries by forming clots. However, unregulated platelet activity can cause inflammation, thrombosis, atherosclerosis, myocardial infarction, and stroke [13].

Hypoglycemia has been associated with increased platelet activity and hence this can promote the development of CVDs.

## 2. Platelet Biology

### 2.1. Thrombopoiesis

Platelets are small anucleate cell fragments that have an array of functions, including hemostasis and immune modulation [16]. The formation of platelets, termed thrombopoiesis, in adults occurs primarily in the bone marrow of long bones. Thrombopoiesis starts with the differentiation of hematopoietic stem cells. Long cytoplasmic processes extend from megakaryocytes into the bone marrow sinusoids, which later shed to form structures called pro-platelets [16]. The pro-platelets then break up into tiny fragments to form platelets which are then released into the circulation. Thrombopoiesis is regulated by thrombopoietin, which is produced by the liver when the platelet count decreases [17]. Secretion of thrombopoietin increases platelet production by increasing the formation of pro-platelets from megakaryocytes [17]. Platelets remain in the circulation for 7 to 10 days until they are eliminated by specialized macrophages in the liver and spleen or utilized to maintain vascular integrity [18].

### 2.2. Platelets in Hemostasis and Thrombosis

**Figure 1:**
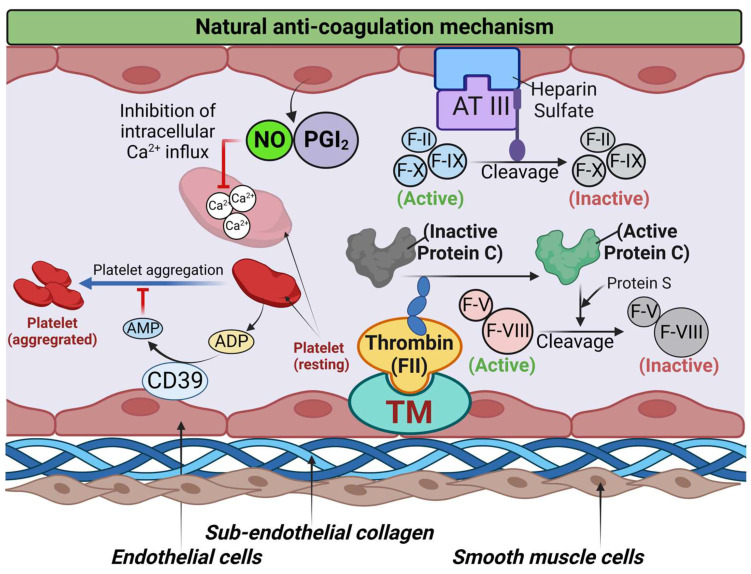
Natural anti-coagulation mechanism. In the normal physiological condition, nitric oxide (NO) and prostacyclin (PGI2) secreted from intact endothelial cells inhibit platelets, keeping them inactive and preventing them from binding to the endothelial lining. NO and prostacyclin act on platelets to inhibit intracellular Ca^2+^ influx in platelets, thus inhibiting the release of granules from platelets and preventing platelet aggregation. Another endothelial cell molecule, CD39 (an adenosine diphosphatase (ADPase)) converts ADP (a potent platelet-derived platelet activator) to adenosine monophosphate (AMP), therefore restricting the availability of ADP to initiate platelet activation followed by platelet aggregation. Coagulation factors are also inactivated by antithrombin III (ATIII), a protein bound to endothelial cells by a glycosaminoglycan, heparin sulfate, and activated protein C (APC) in association with protein S. ATIII cleaves circulating clotting factors (Factor II (F-II), Factor IX (F-IX), and Factor X (F-X)) and inactivates them. Protein C is activated by thrombin (F-II) bound to thrombomodulin (TM) in the endothelial cells. APC cleaves circulating clotting factors (Factor V (F-V), Factor VIII (F-VIII)) and inactivates them.

Platelets are a key mediator of thrombosis (blood clot formation), especially arterial thrombosis, forming platelet-rich white thrombi [19]. Under physiological conditions, the intact endothelium inhibits platelet activation and aggregation by multiple mechanisms as summarized in Figure 1. These mechanisms include an inhibition of the release of platelet granules by prostacyclin and nitric oxide and a breakdown of ADP by the ectonucleotidase CD39. Prostacyclin and nitric oxide increase cAMP and cGMP levels in platelets and restrict the availability of intracellular Ca^2+^ which, in turn, suppresses most platelet activation processes, including adhesion, pseudopod formation, secretion, aggregation, and procoagulant activity [17].

Coagulation factors are also inactivated by antithrombin III (ATIII), a protein bound to endothelial cells by a glycosaminoglycan (heparin sulfate) and activated protein C (APC) in association with protein S. ATIII cleaves circulating clotting factors (Factor II (F-II), Factor IX (F-IX), and Factor X (F-X)) and inactivates them [20]. Protein C is activated by thrombin (F-II) bound to thrombomodulin (TM) in the endothelial cells. APC cleaves circulating clotting factors (Factor V (F-V), Factor VIII (F-VIII)) and inactivates them [21].

**Figure 2:**
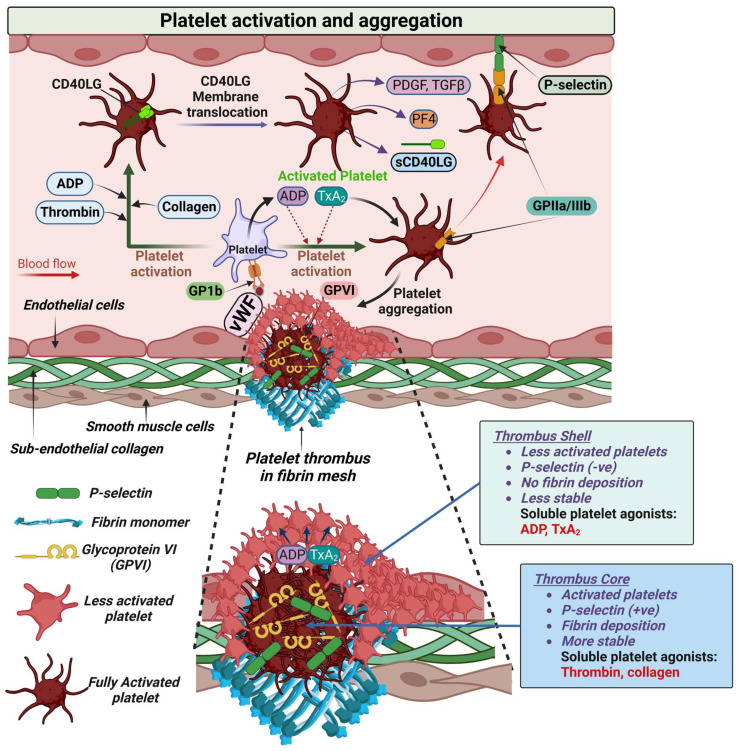
Platelet activation and aggregation. In response to damage to endothelial cells, circulating platelets migrate to the site of injury and bind to the Von Willebrand factor (vWF) protein, that is produced by endothelial cells through another platelet surface protein, glycoprotein Ib (GPIb), and this activates the platelets. Activated platelets release granules containing adenosine di-phosphate (ADP) and thromboxane A2 (TXA2) which bind to their respective receptors expressed on platelets, allowing more platelets to migrate and form clusters at the site of injury; this process is called “platelet aggregation”, and through this process “platelet-thrombus” is formed (in association with fibrin monomer-derived fibrin mesh and glycoprotein VI (GPVI)) at the injury site. Platelet activation also allows the membrane translocation of CD40 ligand (CD40LG). The translocation of CD40LG coincides with the release of α-granule contents, including platelet-derived growth factor (PDGF), transforming growth factor beta (TGFβ), and platelet factor 4 (PF4). The surface-expressed CD40LG is cleaved and shed from the platelet surface in a time-dependent manner as sCD40LG. At the site of injury, platelet–endothelial interaction yields a gradient of platelet thrombus with a core (thrombus core expressing P-selectin) of fully activated platelets overlaid by a shell (thrombus shell) of less activated platelets. Activated platelets release soluble mediators like ADP and TxA2 which act as a positive-feedback mediator of platelet adhesion by increasing the expression of integrin GPIIb/IIIa-receptor and ensuring the rapid activation and recruitment of platelets into the growing thrombus. Inset (between dotted lines), a high-power illustration of ruptured endothelium and the components of platelet thrombus (thrombus core and thrombus shell).

When the integrity of the vascular endothelium is breached by an injury or rupture of an atherosclerotic plaque, these inhibitory mechanisms are lost and platelets are exposed to the subendothelial collagen in the vascular intima and atherosclerotic plaques [17]. Platelets bind to sub-endothelial collagen at the site of endothelial injury via the integrin α_2_β_1_ receptor and Von Willebrand factor released from Weibel Palade bodies via glycoprotein (GP)Ib-IX-V [17]. After binding, platelets are activated and release key mediators of platelet recruitment and aggregation, leading to platelet thrombus formation [17]. Platelet thrombus is an agonist-driven process where one group of platelets (fully activated) form a core overlaid by a shell of less-activated platelets [22]. While the core, which is closest to the site of vascular injury, is characterized by fully activated platelets that express P-selectin, the outer layer of the developing thrombus (thrombus shell) does not express P-selectin. Thromboxane (TxA2), a potent platelet activator, and ADP have proven to be the main soluble agonists of platelet accumulation in the shell (Figure 2). TxA2 or ADP act as positive feedback mediators of platelet adhesion by increasing the expression of integrin GPIIb/IIIa-receptor and ensure the rapid activation and recruitment of platelets into the growing thrombus (Figure 2) [17,23]. These events result in the formation of a temporary platelet plug leading to fibrin formation in secondary hemostasis [19]. Although the physiological function of these processes is to repair the vascular injury, in the setting of atherosclerotic plaque rupture these processes result in the formation of a thrombus responsible for acute occlusion of blood vessels [24].

### 2.3. Platelet Priming

The threshold for platelet activation is not static, the activation threshold of circulating platelets undergoes modulation by numerous factors (Table 1). This phenomenon has led to the emerging concept of platelet priming to explain why, under specific pathological conditions, the platelet activation threshold is increased (negative platelet priming) or decreased (positive platelet priming) [25]. Positive platelet primers can decrease the platelet activation threshold but, on their own, these molecules are not capable of activating platelets. Instead, these molecules just potentiate the activation of platelets.

Positive primers include adrenaline, which acts via the Gαi-coupled α2-adrenergic receptor to decrease platelet cytosolic cAMP levels [25]. Of note, cAMP is an inhibitor of platelet aggregation; therefore, reducing its levels enhances the activity of platelet activators [26]. Insulin-like growth factor I and thrombopoietin are also examples of positive primers [25]. Positive primers can also increase collagen-dependent activation of platelets and thrombus stability [25]. The clinical relevance of the positive primers is that they can offset the action of anti-platelet drugs, such as aspirin and P2Y-12 inhibitors, a cornerstone in treating patients with atherosclerotic disease [17].

Negative platelet-priming substances include biomolecules secreted by endothelial cells, such as prostacyclin and PG E2, which increase cytosolic cAMP levels resulting in an increased threshold for platelet activation [27]. Endothelial cells also form nitric oxide which prevents the activation of platelets and is mediated by an elevation in cytosolic cGMP levels. Other negative platelet primers include adenosine and insulin [27].

In pathological states, the excitability of platelets might be modulated by creating an imbalance between negative and positive priming factors. For instance, in the setting of hypoglycemia, a common side effect of insulin and insulin secretagogues used in the treatment of patients with diabetes [28], the balance between positive and negative primers is acutely offset, resulting in an acutely decreased threshold for platelet activation [29]. Although the effect of hypoglycemia on platelets is short-lived, it is thought that the cumulative effects of the acute decrease in platelet activation threshold are offset due to recurrent hypoglycemic episodes that jeopardize the cardiovascular benefit of tight glycemic control regimens [30,31].

In a study conducted on 12 patients with T2D, activation of platelets by hypoglycemia was reversed when measured 30 min after insulin-induced hypoglycemia [32]. Several mechanisms have been proposed to explain the effect of hypoglycemia on platelets. Studies published in the 1980s have suggested that the secretion of adrenaline in response to hypoglycemia might be responsible for platelet activation by hypoglycemia [33]. However, in a more recent study, decreased platelet sensitivity to prostacyclin was implicated as the mechanism responsible for the decreased platelet activation threshold in the setting of acute hypoglycemia in diabetic patients [34].

## 3. Role of Platelets in Cardiovascular Diseases

Cardiovascular diseases are one of the leading causes of morbidity and mortality in adults worldwide [35] and particularly in T2D [36].

The role of platelets in the development of macrovascular complications of diabetes, such as coronary heart disease, cerebrovascular disease, and peripheral artery disease, has been intensively studied. Besides the well-established role of platelets in acute thrombotic events in the setting of atherosclerotic plaque rupture, platelets play a crucial role in the initiation and progression of atherosclerosis, a key mediator of the macrovascular complications of T2D [16,17,37,38].

## 4. Hypoglycemia and Platelet Dysfunction

Hypoglycemia is associated with platelet reactivity and activation. In 1982, a study on insulin-induced hypoglycemia in juvenile diabetics found that an increase in fibrinogen and Factor VIII reduced activated partial thromboplastin time (PTT) and decreased platelet count to be associated with hypoglycemia [39]. More recently, a randomized controlled trial found that hypoglycemia induces platelet activation and increases platelet-monocyte aggregation and P-selectin (a marker for platelet activation) [40]. Another study on adults with and without T1D found hypoglycemia to be associated with an enhanced expression of proinflammatory mediators, such as plasminogen activator inhibitor 1 (PAI-1), vascular adhesion molecules, intracellular adhesion molecule (ICAM), and E-selectin [41]. The methodology of the latter study maintained hypoglycemia for 120 min compared to the former study that maintained hypoglycemia for 60 min, suggesting that the length of hypoglycemia may play an important part in the platelet response. A study on T2D and control subjects undergoing a hyperinsulinemic clamp found that prothrombotic platelet proteins, namely P-selectin, platelet factor 4, platelet glycoprotein VI, and PAI-1, were elevated in T2D subjects in comparison to control subjects [42], making them more susceptible to thromboembolic events. Induced hypoglycemia in T2D patients was also found to increase platelet activity through reduced sensitivity to prostacyclin, which may lead to increased blood coagulation and thereby increase the risk of CVD [34]. These limited studies suggest that enhanced platelet activation may be multifactorial.

Hypoglycemia causes prothrombotic, platelet proaggregatory and proinflammatory responses that can counter the antiplatelet and anti-inflammatory effects of insulin. Earlier studies recorded an increase in platelet aggregation following insulin-induced hypoglycemia for at least 2 h following the induction of hypoglycemia [43]. However, recent studies have recorded a greater prolonged effect. A study by Chow et al. in 2019 found that hypoglycemia enhances platelet reactivity in patients with and without diabetes up to day 7 following the event, and that this activation occurred with a delay of 24 h [30]. Hypoglycemia in the diabetes group also prolonged clot density and impaired fibrinolysis for at least 7 days compared to the non-diabetes group [30]. Another hypoglycemic clamp study in 2019, in subjects with a moderate hypoglycemic plateau (30 min of a blood glucose of 3.5 mmol/L and 30 min of a blood glucose of 2.5 mmol/L) on metformin therapy and no antiplatelet agents, found an increase in platelet activation, which was evident both at 24 h and at a week after the hypoglycemic episode [44]. Since platelets remain in the circulation for 7 to 10 days, the authors speculated that the sustained platelet activation was due to a subsequent mechanism that lasted longer than the low blood glucose levels [44]. However, these studies were conducted with hypoglycemic clamps in a highly artificial setting, which may not reflect that seen in clinical practice. Nonetheless, these data suggest that, irrespective of hypoglycemia reversal, platelet effects persist much longer than previously recognized and CV events may occur temporally distanced from the initial hypoglycemic event. 

Studies on the mechanisms underlying platelet activation due to hypoglycemia have found counter-regulatory catecholamines responsible for the increase in platelet activation [31,37]. The sympathetic nervous system is activated after hypoglycemia, and counter-regulatory hormones are released to increase blood glucose levels and induce metabolic changes. In a study by Hutton et al., hypoglycemia released adrenaline, a platelet-aggregating agent [37]. Another study found that hypoglycemia does not directly cause platelet hyperactivity [31], but rather increases epinephrine levels, through the activation of α2-adrenergic receptors, causing platelet sensitivity to several aggregating agents to increase in vitro and inducing a platelet release reaction in vivo [31]. Increased plasma epinephrine was also responsible for increased beta-thromboglobulin, a platelet-derived protein [45,46]. These studies emphasize that the effect of hypoglycemia on platelet activation may be both by direct and indirect mechanisms.

Joy et al. reported an increase in non-esterified fatty acids (NEFA) in T2D hypoglycemic studies and hypothesized that this might contribute to hypoglycemia-induced platelet aggregation [47]. An increase in free fatty acid concentration can induce oxidative stress and has a proinflammatory effect evident by the increase in NF-κB binding activity, MIF (a pro-inflammatory cytokine), reactive oxygen species, and p65 expression in circulating mononuclear cells [48]. Previous studies have established that NEFAs can have potential thrombogenic effects, mainly platelet aggregation and the activation of clotting factors [49,50]. Moreover, when platelet-rich plasma was incubated with prostacyclin and albumin-bound fatty acids, prostacyclin inhibited platelet aggregation before exposure to any aggregating agents [51]. The binding of NEFAs to albumin prevents platelet activation [52]; therefore, a reduction in albumin, an increase in NEFAs, or a combination of the two could cause platelet activation [53]—the binding capacity of NEFAs to albumin was reduced by 32% in diabetic subjects [54]. Moreover, platelets incubated with albumin derived from T2D subjects aggregated twice as much as those derived from healthy individuals [55]. NEFAs can also affect the stability of prostacyclin [55]. This could explain why subjects with induced hypoglycemia showed reduced sensitivity to prostacyclin at 24 h [34]. Therefore, increased NEFA levels could contribute mechanistically to hypoglycemia-induced platelet aggregation. 

Hypoglycemia also induces proinflammatory and proatherogenic mechanisms. P-selectin has been identified as a marker of platelet activation [56]. Hypoglycemia increased P-selectin in both T1D subjects and healthy individuals [41]. Similarly, hypoglycemia was found to be a stimulus for higher levels of PAI-1 (a thrombosis risk factor) in healthy subjects and subjects with T1D [41]. This can alter the systemic fibrinolytic balance. Moreover, miRNAs, a class of small noncoding RNAs, can play a role in the pathogenesis of hypoglycemia-induced vascular damage. The expression of several miRNAs was altered up to 7 days following hypoglycemia and the pattern of changes in their expression was similar to biomarkers of platelet activation [57]. The authors speculated that miRNAs are released once platelets are activated following a hypoglycemic episode [57]. These results confirmed previous observations of delayed and consistent platelet activation consequent upon hypoglycemia [57] and suggest that miRNA activation may potentiate or maintain platelet activation.

The endothelium is important in maintaining vascular homeostasis. However, hypoglycemia can lead to increased endothelial dysfunction, even in subjects without glucose intolerance [58]. Increased concentration of proinflammatory cytokines can stimulate inducible nitric oxide synthase expression leading to oxidative stress and consequently endothelial dysfunction [59]. In a study by Joy et al., healthy individuals were exposed to effects of single and repeated episodes of clamped hypoglycemia [60]. In this study, acute hypoglycemia reduced NO-mediated endothelial function. Moreover, repeated episodes of hypoglycemia further impaired vascular function by reducing endogenous and exogenous NO-mediated endothelial function [60]. 

**Figure 3:**
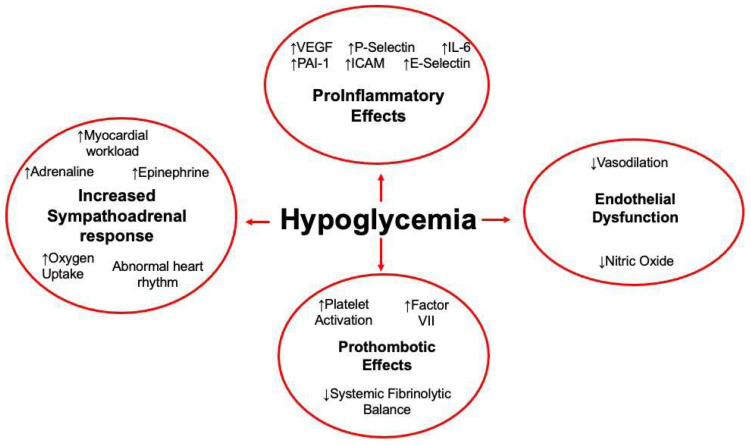
Mechanisms by which hypoglycemia may induce pathophysiological changes in the cardiovascular system.

Hypoglycemia activates different physiological responses, as seen in Figure 3, which may impair vascular function by increasing thrombotic parameters, coagulation biomarkers, inflammatory biomarkers, and inducing platelet activation. Platelets are central to the pathophysiology of CVDs.

## 5. The Association between Hypoglycemia and Cardiovascular Diseases

Studies have found that hypoglycemia is associated with a higher risk of cardiovascular events and mortality [58,61,62]. A meta-analysis of 10 studies showed hypoglycemia to be associated with almost twice the risk of CVD [63]. Hypoglycemia promotes the formation of monocyte platelet aggregates and the interaction between proinflammatory monocytes and platelets [62]. Monocytes can destabilize plaque and rupture, resulting in CVD events [64]. The risk of CVD events has been shown to persist months or years after the hypoglycemic episode [61].

Severe hypoglycemia is one of the stronger predictors of CVD events and has a temporal association with an increased risk of CV events and mortality, especially closer to the episode [65,66,67,68]. However, there is conflicting evidence concerning the association between non-severe hypoglycemia and either CV events or mortality. Non-severe hypoglycemia (self-treated) was not associated with a significant increase in CV events or mortality [65,67]. However, the risk for severe hypoglycemia is increased in individuals experiencing multiple non-severe hypoglycemic episodes [68]. A further study also identified that a high rate of non-severe hypoglycemic episodes was associated with a higher rate of adverse cardiovascular outcomes and severe hypoglycemia in subjects with T2D, a population where this association was previously unclear [69].

Table 2 summarizes the clinical evidence for the association of hypoglycemia and CV outcomes in T2D subjects. A significant concern is the finding that asymptomatic and symptomatic hypoglycemia had the same MACE outcomes and mortality rates [70]. This implies that vascular damage could be induced by non-severe or asymptomatic hypoglycemia and still lead to severe cardiac complications, highlighting the need to address hypoglycemic unawareness.

Considering glycemic control when assessing the effect of severe hypoglycemia on CVD is also important. After stratifying their analyses by the level of hyperglycemia, Fährmann et al. found a cumulative effect of hypoglycemia on coronary artery calcification (CAC) with a clinically relevant risk magnitude of 30% [71]. They found that the effect of hyperglycemia on CAC in patients with poor glycemic control might mask the effect of severe hypoglycemia on CAC [71]. Therefore, they hypothesized that the association between severe hypoglycemia and CVD should be assessed regarding glycemic control [71].

Moreover, acute hypoglycemia and recurrent hypoglycemia can contribute as risk factors for CVD events. Acute hypoglycemia is more likely to be associated with cardiac ischemia and other vascular events than normoglycemia and hyperglycemia [72]. A rapid fall in glucose levels, even within the normal range, led to increased chest pain and ECG abnormalities [72], and among patients with diabetes or with existing vascular damage, acute hypoglycemia may increase the risk of major vascular events, such as myocardial infarction and ischemia [73]. A single hypoglycemic episode can lead to a reduced response to a future hypoglycemic episode and lead to hypoglycemic unawareness leading to recurrent hypoglycemia [72]. Recurrent hypoglycemia has been found to be an aggravating factor in cardiac complications and can lead to a worse prognosis for preclinical atherosclerosis [74]. T1D subjects with recurrent episodes of hypoglycemia were found to have more significant endothelial dysfunction as determined by higher carotid and femoral intima-media thickness, a marker of subclinical atherosclerosis [74]. Continuous glucose monitoring and precautions for intensive insulin therapy are necessary following findings that both repeated and acute occurrences of hypoglycemia can lead to atherosclerosis and CVD events.

Clinical evidence about the relationship between hypoglycemia and CVD risk is less for subjects with T1D, though T1D is considered as one of the cardiovascular risk factors in this population [75]. This is important since hypoglycemia has a higher incidence rate in patients with T1D versus T2D [76]. Despite its potential benefits for managing T1D, insulin therapy increases the risk of hypoglycemia with its potential cardiovascular risk factors [77]. A large prospective study by EURODIAB investigators did not find an increased risk of CVD in subjects with hypoglycemia in T1D [78]; however, other studies have challenged this viewpoint [74,79]. In patients with T1D, cases of nocturnal hypoglycemia have been reported, leading to sudden deaths known as the “death in bed” syndrome and were attributed to cardiac arrhythmia and prolonged QTc interval [80]; however, further research is needed to establish the association between hypoglycemia and CVD in T1D patients. Chronic and elevated inflammatory activity is assumed to cause endothelial damage and increase the risk of CVDs. A recent study found that impaired awareness of hypoglycemia or a history of severe hypoglycemia in T1D subjects was not associated with changes in the inflammatory profile [81]. Overall, current evidence suggests that hypoglycemia affects CVD risk, although the full extent needs to be further studied in the different patient groups.

There are several pathways involved in response to hypoglycemia that lead to adverse cardiovascular outcomes. Catecholamine induction is one of the primary mechanisms by which hypoglycemia induces platelet activation, and catecholamines adversely affect the myocardium and blood vessels. In one study, hypoglycemia decreased vascular endothelial function in subjects without glucose intolerance, and this decrease was correlated with an increase in catecholamine levels following the hypoglycemic stimulus [58]. In an animal study, repeated hypoglycemia caused significantly higher basal adrenaline levels than acute hypoglycemia [82]. These hypoglycemia-induced catecholamines were responsible for the increased adhesion of monocytes to the vascular endothelium, which could lead to vascular endothelium dysfunction [82]. These studies again emphasize that the effect of hypoglycemia on increased CV risk may be both by direct and indirect mechanisms.

Cardiac workload also increases during hypoglycemia which can be dangerous for T2D patients with existing vascular diseases, potentially leading to myocardial ischemia [83]. Furthermore, the association of hypoglycemia with inflammatory markers, such as interleukin (IL)-6 and IL-8, could also lead to endothelial damage and coagulation abnormalities, thereby increasing the risk of CVD events [84]. Certain inflammatory cytokines such as IL-1 can induce a positive feedback cycle, thereby increasing the severity of hypoglycemia [84]. Hypoglycemia has not been directly associated with fatal CVD events because of the lack of simultaneous blood glucose and cardiac monitoring [83], but it has been indirectly associated with CVD events such as myocardial infarctions that may be fatal [83].

The relationship between hypoglycemia and CVD is complex. For example, studies have suggested that the association between severe hypoglycemia and CVD can be a marker of frailty because comorbidity may increase the risk of hypoglycemia, and hypoglycemia in this population can lead to poor outcomes [69,85]. However, the existing evidence strongly indicates hypoglycemia as one of the risk markers and risk factors for CVD. Diabetic patients who need insulin therapy or oral sulfonylureas and individuals at high cardiovascular risk should be given a treatment plan that minimizes the risk of hypoglycemia and its associated CVD risk factors. Furthermore, measures such as continuous glucose monitoring should be employed to help identify and avoid severe hypoglycemia.

## 6. Therapies for the Prevention of Cardiovascular Disease

A primary goal in treating diabetes is to optimize glycemic control to reduce the risk of cardiovascular events [86]. Previously, it was hypothesized that tight glycemic control might reduce the risk of CVD; however, conversely, the clinical evidence shows that tight glycemic control in patients with diabetes comes at the cost of increasing the risk of hypoglycemia and increased mortality through increasing the risk of CVDs [87]. Two extensive meta-analysis studies evaluated the association between hypoglycemia and CVD in diabetic patients. One study concluded that confounding comorbidities alone cannot explain the identified association as the prevalence of the comorbidities is not of a sufficient magnitude to fully account for the detected association [63]. In the second, a dose–response relationship was found between hypoglycemia and the risk of CVD, implying a causal relationship between hypoglycemia and the risk of CVD [88]. Therefore, minimizing the risk of hypoglycemia is thought to be beneficial for reducing the risk of CVDs in patients with diabetes. This can be achieved by using therapeutic strategies associated with a low risk of hypoglycemia. Such approaches are likely to prove most beneficial for patients such as those with hypoglycemia unawareness as a result of cognitive impairment or hypoglycemia-induced autonomic failure, and patients with comorbidities such as renal impairment and liver failure [86,89].

These strategies are also likely to be particularly beneficial for patients at an early stage of diabetes since they allow for intensive glycemic control with a minimal risk of hypoglycemia. Tight glycemic control early in the course of diabetes is likely to reduce the risk of microvascular and macrovascular complications of diabetes later in life, regardless of glycemic control in the later course of diabetes owing to the metabolic memory legacy effect [90].

The following are evidence-based approaches aimed at reducing the risk of hypoglycemia and subsequently reducing the risk of CVDs.

### 6.1. Avoiding the Use of Sulfonylureas

In patients with T2D, a reduction in hypoglycemia risk may be achieved by using a medication regimen that does not include sulfonylureas. This approach might allow the cardiovascular benefits of lowering HbA1c without increasing the risk of hypoglycemia [91]. However, in clinical practice, avoiding sulfonylureas might not always be practical for reasons including financial constraints and formulary restrictions [92].

Sulfonylureas have long been linked to an increased risk of CVD compared to other oral hypoglycemics; however, the cause of this association is debated [93]. In a large retrospective observational study of 77,138 diabetic patients treated with metformin between 1998 and 2013, switching to or adding a sulfonylurea (with mean follow-up of 1.1 years) resulted in an increased risk of myocardial infarction (HR 1.26, 95% CI 1.01–1.56) and all-cause mortality (HR 1.28, 95% CI 1.15–1.44), compared with continuing metformin monotherapy [94]. Severe hypoglycemia occurred more frequently in the group treated with sulfonylureas [94]. Of note, the study participants were matched for HbA1c to eliminate the confounding effect of HbA1c on cardiovascular mortality.

Therefore, it is possible that the higher rate of severe hypoglycemia among the patients treated with sulfonylureas could be responsible for the excess incidence of myocardial infarction and all-cause mortality in the sulfonylurea group. Nevertheless, the study is observational and is therefore inherently limited by confounding factors. For instance, weight gain associated with using sulfonylureas may have confounded the results. However, the increase in the risk of CVD was even seen in patients who used sulfonylureas for a short duration, suggesting that a more acute event such as hypoglycemia was responsible for the observed association [94].

Time lag bias might have also confounded the results of this study. Sulfonylureas are usually started as an add-on therapy in diabetes treatment. Therefore, it is possible that starting sulfonylureas at a more advanced stage of diabetes results in a higher rate of CVDs and mortality in the sulfonylurea group. In a methodological review of observational studies comparing sulfonylureas to metformin, time lag bias was cited as an important factor skewing the results of such studies [95]. Due to the possible cardiovascular risk of sulfonylureas, the current American and European guidelines recommend that sulfonylureas only be used when other oral diabetes drugs with proven cardiovascular benefits, such as metformin, SGLT2i, glucagon-like peptide-1 receptor agonists, or DPP4i, cannot achieve the target HbA1c [96]. Of note, these classes of drugs are associated with a lower risk of hypoglycemia than sulfonylureas [96], a factor perhaps responsible for the more favorable cardiovascular profile of these other classes of oral hypoglycemic drugs.

### 6.2. New Insulin Preparations

Insulin is required for patients with T1D and indicated for patients with advanced T2D whose hyperglycemia does not respond to oral hypoglycemic medications [96,97]. Insulin is available in both long-acting and short-acting forms. Short-acting forms provide rapid but short-lived glycemic control after meals, but long-acting forms offer coverage throughout the day. Of the long-acting forms of insulin, degludec has a lower risk of hypoglycemia, possibly due to its longer duration of action and lesser day-to-day variability in absorption [98,99]. It is possible that the reduced risk of hypoglycemia with insulin degludec might translate into a lower rate of CVDs. In a meta- analysis of randomized controlled trials, insulin degludec was associated with a lower rate of CVD compared to insulin glargine despite equivalent HbA1c with both types of insulin [100]. Therefore, it is possible that the reduced risk of hypoglycemia in patients treated with insulin degludec could be responsible for the observed reduction in the risk of CVD.

### 6.3. Altering the Method of Insulin Delivery

In patients with T1D, the risk of hypoglycemia can be reduced by using an insulin pump for insulin delivery instead of multiple daily injections. This approach permits a reduced risk of hypoglycemia without sacrificing glycemic control [96]. The reduction in the risk of hypoglycemia is most significant with hybrid closed-loop systems, especially in systems with threshold suspend features [101]. A novel bi-hormonal pump delivering insulin and glucagon currently under investigation was found to have a lower risk of hypoglycemia than the currently used insulin pumps; however, this pump is not yet commercially available [102]. Studies consistently show a reduced risk of CVD with insulin pumps. However, from the current studies, it is unclear whether the observed reduction is due to better glycemic control or reduced risk of hypoglycemia [103].

### 6.4. Individualizing HbA1c Targets

The current European and American guidelines suggest that HbA1c targets should be individualized so that more liberal HbA1c targets are utilized for patients at higher risk of hypoglycemia (examples being elderly patients and patients with impaired hypoglycemia awareness) and for patients with severe or frequent hypoglycemia [104,105]. These recommendations are backed by evidence from landmark clinical trials of glycemic control targets [9,106,107]. What is not currently clear is whether in younger patients with recent onset diabetes, who have not yet developed cardiovascular complications of diabetes, the mortality benefit of the reduced future risk of developing cardiovascular complications with tighter glycemic control regimens due to the metabolic memory effect would outweigh the risks associated with hypoglycemia [90]. The patient cohort in the ACCORD study, which demonstrated excess mortality with tight glycemic control, had a mean age of 62.2 years and a median duration of diabetes of 10 years [9]. Almost 35% of the patient cohort had already had at least one cardiovascular event at baseline [9]. In the ADVANCE and the VADT study, which demonstrated that tight glycemic control does not affect mortality, the median cohort age was 66 and 60.3 years, respectively, the median diabetes duration was 7.9 years and 11.5 years, respectively, and the percentage of patients who had cardiovascular events at baseline was 32.2% and around 40%, respectively [106,107] (Table 3). Given the patient demographics in these studies, the results might not apply to younger patients with recent-onset diabetes. Future studies are needed to investigate the long-term effect of tight glycemic control in younger patients.

## 7. Future Directions and Perspectives 

The current consensus is that hypoglycemia leads to an increase in CVD risk. Platelets likely play an under-recognized role in the pathogenesis of hypoglycemia induced CVDs. Clinical studies are needed to investigate whether the proposed methods of reducing the risk of hypoglycemia decrease the risk of CVDs. The main limitation is that the current studies have only been performed on a limited cohort of patients with hypoglycemia.

Most work has focused upon hypoglycemic clamp studies, which use static blood glucose levels and therefore may not reflect the normal clinical scenario. The rates of change in blood glucose that occur during a hypoglycemic episode might also have an impact on platelet activation. Moreover, more studies using flow-cytometry-based platelet activation assays instead of light aggregometry as endpoints are also needed to sensitively record the changes observed during hypoglycemia. 

The current studies reported use hypoglycemic levels that are well below the threshold for the definition of hypoglycemia. Studies with incrementally decreasing “marginal hypoglycemic” targets would be useful for identifying the serum glucose threshold at which the activation of platelets occurs. These would also help guide the optimal glycemic treatment targets for patients with diabetes or those with existing cardiovascular complications of diabetes.

Current studies focus on the effects of a single episode of hypoglycemia; however, the cumulative effects of recurrent hypoglycemia on inflammation, platelet activation, and endothelial dysfunction are largely unknown. 

Although there is ample evidence from laboratory-based studies to support the role of platelets in hypoglycemia-induced CVDs, the clinical effectiveness of antiplatelet drugs in preventing hypoglycemia-induced CVDs remains uncertain. To date, only two post- analysis studies were performed using data from the ACCORD and ADVANCE studies to investigate this association [106,109]. In the post hoc analysis of the ACCORD study, the use of antiplatelet drugs was associated with increased mortality risk [106]. In the ADVANCE study, the antiplatelet drug aspirin did not significantly affect mortality [109]. Although the results of these studies do not support the use of antiplatelet drugs as primary prevention for hypoglycemia-induced CVD, they do not necessarily translate to a lack of efficacy of antiplatelet drugs in CVD protection in hypoglycemia. Future clinical studies are needed to answer this crucial question. 

## 8. Clinical Implications

This review may serve to guide future studies on the utility of antiplatelet drugs for the prevention of hypoglycemia-induced CVDs. The identification of such a treatment that protects against hypoglycemia-induced CVD could lead to its use as an adjunct therapy in vulnerable patients and help facilitate optimal glycemic control to prevent the microvascular complications of diabetes without increasing the risk of CVD. In the ACCORD eye study, after a follow up period of 4 years, intensive glycemic control significantly reduced the risk of retinopathy progression (OR = 0.67; 95% CI, 0.51–0.87; *p* = 0.003) [108]. In addition, in the ADVANCE study, intensive glycemic control resulted in a significant reduction in the risk of new or worsening nephropathy (HR = 0.79; 95% CI 0.66–0.93; *p* = 0.006) [106]. These results show that optimal glycemic control is beneficial in reducing the risk of microvascular complications of diabetes but is currently at risk of inducing hypoglycemia.

## 9. Conclusions

The existing evidence indicates that hypoglycemia is a risk factor for CVDs, and symptomatic and asymptomatic hypoglycemia can both lead to adverse cardiovascular outcomes in T1D and T2D subjects. Hypoglycemia has been associated with prothrombotic, platelet proaggregatory, and proinflammatory responses leading to a potential hypercoagulable state. The increase in platelet activation is due to counter-regulatory catecholamines, NEFAs, and other proteins, such as P-selectin and PA-I. Optimal glycemic control is key in preventing microvascular diabetes-related complications and reducing the risk of CVDs, whilst minimizing the risk of hypoglycemia. Further research is needed to clarify the mechanisms of platelet activation induced by hypoglycemia and the impact of hypoglycemia on CVDs. In addition, clinical studies are needed to investigate the potential clinical effectiveness of antiplatelet drugs in preventing hypoglycemia-induced CVDs, which may facilitate optimal glycemic control for the prevention of microvascular complications of diabetes without increasing the risk of CVD.

## Figures and Tables

**Table 1:** A summary of initiators of platelet activation and platelet primers.

Platelets		
Initiators of Platelet Activation	Positive Primers	Negative Primers
Subendothelial collagen	Adrenaline	Nitric oxide
Von Willebrand Factor	Insulin-like Growth Factor 1	Prostacyclin
Fibrinogen	Thrombopoietin	Adenosine
Laminin	GAS6	Insulin
Thrombin	SCD40L	PGE2

**Table 2:** Clinical studies related to the association of hypoglycemia with CV outcomes in type 2 diabetic subjects.

Study	Year	Sample Size	Follow-Up Period	Type of Diabetes	Definition of Hypoglycemia	Effect Size
DEVOTE 3 [66]	2018	7637	Median 2.0 years	Type 2	Severe hypoglycemia	All-cause mortality HR 2.51(1.79–3.50) CVD HR 1.38(0.96–1.96)
ORIGIN [67]	2013	12,537	Median 6.2 years	Type 2	Severe hypoglycemia	Primary outcomes HR 1.58(1.24–2.02) Mortality HR 1.74(1.39–2.19) CV Death HR1.71(1.27–2.30) Arrhythmic death HR 1.77(1.17–2.67)
ORIGIN [67]	2013	12,537	Median 6.2 years	Type 2	Non-severe hypoglycemia	No association

Abbreviation: HR, Hazard Ratio.

**Table 3:** Population demographics of patient cohorts in the ACCORD, ADVANCE, and VADT studies.

Study	Mean Cohort Age	Median Duration of Diabetes (Years)	Patients Diagnosed with One or More CVD at Baseline (%)
ACCORD [108]	62.2	10	35
ADVANCE [106]	60	7.9	32.2
VADT [107]	60.3	11.5	40

## Data Availability

Not applicable.

## References

[1] Mathew P., Thoppil D. (2022). Hypoglycemia. StatPearls.

[2] American Diabetes Association (2020). Glycemic Targets: Standards of Medical Care in Diabetes—2020. Diabetes Care.

[3] Henriksen M.M., Andersen H.U., Thorsteinsson B., Pedersen-Bjergaard U. (2018). Hypoglycemic Exposure and Risk of Asymptomatic Hypoglycemia in Type 1 Diabetes Assessed by Continuous Glucose Monitoring. J. Clin. Endocrinol. Metab..

[4] Heller S.R., Peyrot M., Oates S.K., Taylor A.D. (2020). Hypoglycemia in patient with type 2 diabetes treated with insulin: It can happen. BMJ Open Diabetes Res. Care.

[5] Melo K.F.S., Bahia L.R., Pasinato B., Porfirio G.J.M., Martimbianco A.L., Riera R., Calliari L.E.P., Minicucci W.J., Turatti L.A.A., Pedrosa H.C. (2019). Short-acting insulin analogues versus regular human insulin on postprandial glucose and hypoglycemia in type 1 diabetes mellitus: A systematic review and meta-analysis. Diabetol. Metab. Syndr..

[6] Oyer D.S. (2013). The science of hypoglycemia in patients with diabetes. Curr. Diabetes Rev..

[7] Nirantharakumar K., Marshall T., Hodson J., Narendran P., Deeks J., Coleman J.J., Ferner R.E. (2012). Hypoglycemia in non-diabetic in-patients: Clinical or criminal?. PLoS ONE.

[8] Tsujimoto T., Yamamoto-Honda R., Kajio H., Kishimoto M., Noto H., Hachiya R., Kimura A., Kakei M., Noda M. (2015). Prediction of 90-day mortality in patients without diabetes by severe hypoglycemia: Blood glucose level as a novel marker of severity of underlying disease. Acta Diabetol..

[9] Gerstein H.C., Miller M.E., Byington R.P., Goff D.C., Bigger J.T., Buse J.B., Cushman W.C., Genuth S., Ismail-Beigi F., Grimm R.H. (2008). Effects of intensive glucose lowering in type 2 diabetes. N. Engl. J. Med..

[10] Sanon V.P., Sanon S., Kanakia R., Yu H., Araj F., Oliveros R., Chilton R. (2014). Hypoglycemia from a cardiologist’s perspective. Clin. Cardiol..

[11] Moin A.S.M., Sathyapalan T., Atkin S.L., Butler A.E. (2022). The severity and duration of Hypoglycemia affect platelet-derived protein responses in Caucasians. Cardiovasc. Diabetol..

[12] Kahal H., Halama A., Aburima A., Bhagwat A.M., Butler A.E., Graumann J., Suhre K., Sathyapalan T., Atkin S.L. (2020). Effect of induced hypoglycemia on inflammation and oxidative stress in type 2 diabetes and control subjects. Sci. Rep..

[13] Lebas H., Yahiaoui K., Martos R., Boulaftali Y. (2019). Platelets Are at the Nexus of Vascular Diseases. Front. Cardiovasc. Med..

[14] Aliotta A., Bertaggia Calderara D., Zermatten M.G., Alberio L. (2021). High-Dose Epinephrine Enhances Platelet Aggregation at the Expense of Procoagulant Activity. Thromb. Haemost..

[15] von Känel R., Heimgartner N., Stutz M., Zuccarella-Hackl C., Hänsel A., Ehlert U., Wirtz P.H. (2019). Prothrombotic response to norepinephrine infusion, mimicking norepinephrine stress-reactivity effects, is partly mediated by α-adrenergic mechanisms. Psychoneuroendocrinology.

[16] Tyagi T., Jain K., Gu S.X., Qiu M., Gu V.W., Melchinger H., Rinder H., Martin K.A., Gardiner E.E., Lee A.I. (2022). A guide to molecular and functional investigations of platelets to bridge basic and clinical sciences. Nat. Cardiovasc. Res..

[17] van der Meijden P.E.J., Heemskerk J.W.M. (2019). Platelet biology and functions: New concepts and clinical perspectives. Nat. Rev. Cardiol..

[18] Quach M.E., Chen W., Li R. (2018). Mechanisms of platelet clearance and translation to improve platelet storage. Blood.

[19] Koupenova M., Kehrel B.E., Corkrey H.A., Freedman J.E. (2017). Thrombosis and platelets: An update. Eur. Heart J..

[20] Versteeg H.H., Heemskerk J.W., Levi M., Reitsma P.H. (2013). New fundamentals in hemostasis. Physiol. Rev..

[21] Dahlbäck B., Villoutreix B.O. (2005). The anticoagulant protein C pathway. FEBS Lett..

[22] Brass L.F., Tomaiuolo M., Welsh J., Poventud-Fuentes I., Zhu L., Diamond S.L., Stalker T.J., Michelson A.D. (2019). 20—Hemostatic Thrombus Formation in Flowing Blood. Platelets.

[23] Offermanns S. (2006). Activation of platelet function through G protein-coupled receptors. Circ. Res..

[24] Lippi G., Franchini M., Targher G. (2011). Arterial thrombus formation in cardiovascular disease. Nat. Rev. Cardiol..

[25] Baaten C., Ten Cate H., van der Meijden P.E.J., Heemskerk J.W.M. (2017). Platelet populations and priming in hematological diseases. Blood Rev..

[26] Raslan Z., Naseem K.M. (2014). The control of blood platelets by cAMP signalling. Biochem. Soc. Trans..

[27] Stefanini L., Bergmeier W. (2018). Negative regulators of platelet activation and adhesion. J. Thromb. Haemost..

[28] Frier B.M. (2014). Hypoglycaemia in diabetes mellitus: Epidemiology and clinical implications. Nat. Rev. Endocrinol..

[29] Papazafiropoulou A., Papanas N., Pappas S., Maltezos E., Mikhailidis D.P. (2015). Effects of oral hypoglycemic agents on platelet function. J. Diabetes Complicat..

[30] Dandona P., Chaudhuri A., Dhindsa S. (2010). Proinflammatory and prothrombotic effects of hypoglycemia. Diabetes Care.

[31] Yamamoto K., Ito T., Nagasato T., Shinnakasu A., Kurano M., Arimura A., Arimura H., Hashiguchi H., Deguchi T., Maruyama I. (2019). Effects of glycemic control and hypoglycemia on Thrombus formation assessed using automated microchip flow chamber system: An exploratory observational study. Thromb. J..

[32] Chow E., Iqbal A., Walkinshaw E., Phoenix F., Macdonald I.A., Storey R.F., Ajjan R., Heller S.R. (2018). Prolonged Prothrombotic Effects of Antecedent Hypoglycemia in Individuals with Type 2 Diabetes. Diabetes Care.

[33] Trovati M., Anfossi G., Cavalot F., Vitali S., Massucco P., Mularoni E., Schinco P., Tamponi G., Emanuelli G. (1986). Studies on mechanisms involved in hypoglycemia-induced platelet activation. Diabetes.

[34] Kahal H., Aburima A., Spurgeon B., Wraith K.S., Rigby A.S., Sathyapalan T., Kilpatrick E.S., Naseem K.M., Atkin S.L. (2018). Platelet function following induced hypoglycaemia in type 2 diabetes. Diabetes Metab..

[35] Dong C., Bu X., Liu J., Wei L., Ma A., Wang T. (2022). Cardiovascular disease burden attributable to dietary risk factors from 1990 to 2019: A systematic analysis of the Global Burden of Disease study. Nutr. Metab. Cardiovasc. Dis..

[36] Leon B.M., Maddox T.M. (2015). Diabetes and cardiovascular disease: Epidemiology, biological mechanisms, treatment recommendations and future research. World J. Diabetes.

[37] Lievens D., von Hundelshausen P. (2011). Platelets in atherosclerosis. Thromb. Haemost..

[38] Chatterjee M., Gawaz M. Platelets in Atherosclerosis.

[39] Dalsgaard-Nielsen J., Madsbad S., Hilsted J. (1982). Changes in platelet function, blood coagulation and fibrinolysis during insulin-induced hypoglycaemia in juvenile diabetics and normal subjects. Thromb. Haemost..

[40] Wright R.J., Newby D.E., Stirling D., Ludlam C.A., Macdonald I.A., Frier B.M. (2010). Effects of acute insulin-induced hypoglycemia on indices of inflammation: Putative mechanism for aggravating vascular disease in diabetes. Diabetes Care.

[41] Gogitidze Joy N., Hedrington M.S., Briscoe V.J., Tate D.B., Ertl A.C., Davis S.N. (2010). Effects of acute hypoglycemia on inflammatory and pro-atherothrombotic biomarkers in individuals with type 1 diabetes and healthy individuals. Diabetes Care.

[42] Moin A.S.M., Al-Qaissi A., Sathyapalan T., Atkin S.L., Butler A.E. (2021). Platelet Protein-Related Abnormalities in Response to Acute Hypoglycemia in Type 2 Diabetes. Front. Endocrinol..

[43] Hutton R.A., Mikhailidis D., Dormandy K.M., Ginsburg J. (1979). Platelet aggregation studies during transient hypoglycaemia: A potential method for evaluating platelet function. J. Clin. Pathol..

[44] Aberer F., Pferschy P.N., Tripolt N.J., Sourij C., Obermayer A.M., Prüller F., Novak E., Reitbauer P., Kojzar H., Prietl B. (2020). Hypoglycaemia leads to a delayed increase in platelet and coagulation activation markers in people with type 2 diabetes treated with metformin only: Results from a stepwise hypoglycaemic clamp study. Diabetes Obes. Metab..

[45] Kjeldsen S.E., Gjesdal K., Eide I., Aakesson I., Amundsen R., Foss O.P., Leren P. (1983). Increased beta-thromboglobulin in essential hypertension: Interactions between arterial plasma adrenaline, platelet function and blood lipids. Acta Med. Scand..

[46] Lande K., Gjesdal K., Fønstelien E., Kjeldsen S.E., Eide I. (1985). Effects of adrenaline infusion on platelet number, volume and release reaction. Thromb. Haemost..

[47] Joy N.G., Mikeladze M., Younk L.M., Tate D.B., Davis S.N. (2016). Effects of equivalent sympathetic activation during hypoglycemia on endothelial function and pro-atherothrombotic balance in healthy individuals and obese standard treated type 2 diabetes. Metabolism.

[48] Tripathy D., Mohanty P., Dhindsa S., Syed T., Ghanim H., Aljada A., Dandona P. (2003). Elevation of free fatty acids induces inflammation and impairs vascular reactivity in healthy subjects. Diabetes.

[49] Hoak J.C., Warner E.D., Connor W.E. (1967). Platelets, fatty acids and thrombosis. Circ. Res..

[50] Burstein Y., Berns L., Heldenberg D., Kahn Y., Werbin B.Z., Tamir I. (1978). Increase in platelet aggregation following a rise in plasma free fatty acids. Am. J. Hematol..

[51] Nordøy A., Svensson B. (1979). The simultaneous effect of albumin bound fatty acids on platelets and endothelial cells. Thromb. Res..

[52] Nordøy A. (1979). Albumin-bound fatty acids, platelets and endothelial cells in thrombogenesis. Haemostasis.

[53] Barbano B., Gigante A., Amoroso A., Cianci R. (2013). Thrombosis in nephrotic syndrome. Semin. Thromb. Hemost..

[54] Dhindsa S., Ghanim H., Dandona P. (2015). Nonesterified Fatty Acids, Albumin, and Platelet Aggregation. Diabetes.

[55] Blache D., Bourdon E., Salloignon P., Lucchi G., Ducoroy P., Petit J.M., Verges B., Lagrost L. (2015). Glycated albumin with loss of fatty acid binding capacity contributes to enhanced arachidonate oxygenation and platelet hyperactivity: Relevance in patients with type 2 diabetes. Diabetes.

[56] Ferroni P., Martini F., Riondino S., La Farina F., Magnapera A., Ciatti F., Guadagni F. (2009). Soluble P-selectin as a marker of in vivo platelet activation. Clin. Chim. Acta.

[57] Eyileten C., Wicik Z., Keshwani D., Aziz F., Aberer F., Pferschy P.N., Tripolt N.J., Sourij C., Prietl B., Prüller F. (2022). Alteration of circulating platelet-related and diabetes-related microRNAs in individuals with type 2 diabetes mellitus: A stepwise hypoglycaemic clamp study. Cardiovasc. Diabetol..

[58] Tanaka K., Okada Y., Torimoto K., Nishio K., Narisawa M., Tanaka Y. (2022). Hypoglycemia induces vascular endothelial dysfunction in subjects with normal glucose tolerance. Sci. Rep..

[59] Nagareddy P.R., Xia Z., McNeill J.H., MacLeod K.M. (2005). Increased expression of iNOS is associated with endothelial dysfunction and impaired pressor responsiveness in streptozotocin-induced diabetes. Am. J. Physiol. Heart Circ. Physiol..

[60] Joy N.G., Tate D.B., Younk L.M., Davis S.N. (2015). Effects of Acute and Antecedent Hypoglycemia on Endothelial Function and Markers of Atherothrombotic Balance in Healthy Humans. Diabetes.

[61] Khunti K., Davies M., Majeed A., Thorsted B.L., Wolden M.L., Paul S.K. (2015). Hypoglycemia and risk of cardiovascular disease and all-cause mortality in insulin-treated people with type 1 and type 2 diabetes: A cohort study. Diabetes Care.

[62] Iqbal A., Prince L.R., Novodvorsky P., Bernjak A., Thomas M.R., Birch L., Lambert D., Kay L.J., Wright F.J., Macdonald I.A. (2019). Effect of Hypoglycemia on Inflammatory Responses and the Response to Low-Dose Endotoxemia in Humans. J. Clin. Endocrinol. Metab..

[63] Goto A., Arah O.A., Goto M., Terauchi Y., Noda M. (2013). Severe hypoglycaemia and cardiovascular disease: Systematic review and meta-analysis with bias analysis. BMJ Br. Med. J..

[64] Newby A.C., George S.J., Ismail Y., Johnson J.L., Sala-Newby G.B., Thomas A.C. (2009). Vulnerable atherosclerotic plaque metalloproteinases and foam cell phenotypes. Thromb. Haemost..

[65] Zinman B., Marso S.P., Christiansen E., Calanna S., Rasmussen S., Buse J.B. (2018). Hypoglycemia, Cardiovascular Outcomes, and Death: The LEADER Experience. Diabetes Care.

[66] Pieber T.R., Marso S.P., McGuire D.K., Zinman B., Poulter N.R., Emerson S.S., Pratley R.E., Woo V., Heller S., Lange M. (2018). DEVOTE 3: Temporal relationships between severe hypoglycaemia, cardiovascular outcomes and mortality. Diabetologia.

[67] Mellbin L.G., Rydén L., Riddle M.C., Probstfield J., Rosenstock J., Díaz R., Yusuf S., Gerstein H.C. (2013). Does hypoglycaemia increase the risk of cardiovascular events? A report from the ORIGIN trial. Eur. Heart J..

[68] Festa A., Heller S.R., Seaquist E., Duan R., Hadjiyianni I., Fu H. (2017). Association between mild and severe hypoglycemia in people with type 2 diabetes initiating insulin. J. Diabetes Complicat..

[69] Heller S.R., Geybels M.S., Iqbal A., Liu L., Wagner L., Chow E. (2022). A higher non-severe hypoglycaemia rate is associated with an increased risk of subsequent severe hypoglycaemia and major adverse cardiovascular events in individuals with type 2 diabetes in the LEADER study. Diabetologia.

[70] Wei W., Zhao S., Fu S.-L., Yi L., Mao H., Tan Q., Xu P., Yang G.-L. (2019). The Association of Hypoglycemia Assessed by Continuous Glucose Monitoring WITH Cardiovascular Outcomes and Mortality in Patients with Type 2 Diabetes. Front. Endocrinol..

[71] Fährmann E.R., Adkins L., Loader C.J., Han H., Rice K.M., Denvir J., Driscoll H.K. (2015). Severe hypoglycemia and coronary artery calcification during the diabetes control and complications trial/epidemiology of diabetes interventions and complications (DCCT/EDIC) study. Diabetes Res. Clin. Pract..

[72] Desouza C., Salazar H., Cheong B., Murgo J., Fonseca V. (2003). Association of hypoglycemia and cardiac ischemia: A study based on continuous monitoring. Diabetes Care.

[73] Wright R.J., Frier B.M. (2008). Vascular disease and diabetes: Is hypoglycaemia an aggravating factor?. Diabetes Metab. Res. Rev..

[74] Giménez M., Gilabert R., Monteagudo J., Alonso A., Casamitjana R., Paré C., Conget I. (2011). Repeated episodes of hypoglycemia as a potential aggravating factor for preclinical atherosclerosis in subjects with type 1 diabetes. Diabetes Care.

[75] Vergès B. (2020). Cardiovascular disease in type 1 diabetes: A review of epidemiological data and underlying mechanisms. Diabetes Metab..

[76] Donnelly L.A., Morris A.D., Frier B.M., Ellis J.D., Donnan P.T., Durrant R., Band M.M., Reekie G., Leese G.P. (2005). Frequency and predictors of hypoglycaemia in Type 1 and insulin-treated Type 2 diabetes: A population-based study. Diabet. Med..

[77] Nathan D.M., Genuth S., Lachin J., Cleary P., Crofford O., Davis M., Rand L., Siebert C. (1993). The effect of intensive treatment of diabetes on the development and progression of long-term complications in insulin-dependent diabetes mellitus. N. Engl. J. Med..

[78] Gruden G., Barutta F., Chaturvedi N., Schalkwijk C., Stehouwer C.D., Witte D.R., Fuller J.H., Perin P.C., Bruno G. (2012). Severe hypoglycemia and cardiovascular disease incidence in type 1 diabetes: The EURODIAB Prospective Complications Study. Diabetes Care.

[79] Giménez M., López J.J., Castell C., Conget I. (2012). Hypoglycaemia and cardiovascular disease in Type 1 Diabetes. Results from the Catalan National Public Health registry on insulin pump therapy. Diabetes Res. Clin. Pract..

[80] Gill G., Woodward A., Casson I., Weston P. (2008). Cardiac arrhythmia and nocturnal hypoglycaemia in type 1 diabetes—The ‘dead in bed’ syndrome revisited. Diabetologia.

[81] Ali N., Janssen A.W.M., Jaeger M., Van de Wijer L., van der Heijden W., Ter Horst R., Vart P., van Gool A., Joosten L.A.B., Netea M.G. (2020). Limited impact of impaired awareness of hypoglycaemia and severe hypoglycaemia on the inflammatory profile of people with type 1 diabetes. Diabetes Obes. Metab..

[82] Jin W.L., Azuma K., Mita T., Goto H., Kanazawa A., Shimizu T., Ikeda F., Fujitani Y., Hirose T., Kawamori R. (2011). Repetitive hypoglycaemia increases serum adrenaline and induces monocyte adhesion to the endothelium in rat thoracic aorta. Diabetologia.

[83] Kalra S., Mukherjee J.J., Venkataraman S., Bantwal G., Shaikh S., Saboo B., Das A.K., Ramachandran A. (2013). Hypoglycemia: The neglected complication. Indian J. Endocrinol. Metab..

[84] Chopra S., Kewal A. (2012). Does hypoglycemia cause cardiovascular events?. Indian J. Endocrinol. Metab..

[85] Goto A., Goto M., Terauchi Y., Yamaguchi N., Noda M. (2016). Association Between Severe Hypoglycemia and Cardiovascular Disease Risk in Japanese Patients with Type 2 Diabetes. J. Am. Heart. Assoc..

[86] Seaquist E.R., Anderson J., Childs B., Cryer P., Dagogo-Jack S., Fish L., Heller S.R., Rodriguez H., Rosenzweig J., Vigersky R. (2013). Hypoglycemia and diabetes: A report of a workgroup of the American Diabetes Association and the Endocrine Society. Diabetes Care.

[87] Mannucci E., Dicembrini I., Lauria A., Pozzilli P. (2013). Is glucose control important for prevention of cardiovascular disease in diabetes?. Diabetes Care.

[88] Yeh J.S., Sung S.H., Huang H.M., Yang H.L., You L.K., Chuang S.Y., Huang P.C., Hsu P.F., Cheng H.M., Chen C.H. (2016). Hypoglycemia and risk of vascular events and mortality: A systematic review and meta-analysis. Acta Diabetol..

[89] Cryer P.E. (2013). Mechanisms of hypoglycemia-associated autonomic failure in diabetes. N. Engl. J. Med..

[90] Lachin J.M., Nathan D.M. (2021). Understanding Metabolic Memory: The Prolonged Influence of Glycemia During the Diabetes Control and Complications Trial (DCCT) on Future Risks of Complications During the Study of the Epidemiology of Diabetes Interventions and Complications (EDIC). Diabetes Care.

[91] Lash R.W., Lucas D.O., Illes J. (2018). Preventing Hypoglycemia in Type 2 Diabetes. J. Clin. Endocrinol. Metab..

[92] Silbert R., Salcido-Montenegro A., Rodriguez-Gutierrez R., Katabi A., McCoy R.G. (2018). Hypoglycemia Among Patients with Type 2 Diabetes: Epidemiology, Risk Factors, and Prevention Strategies. Curr. Diab. Rep..

[93] Leiter L.A. (2020). Latest Evidence on Sulfonylureas: What’s New?. Diabetes Ther..

[94] Douros A., Dell’Aniello S., Yu O.H.Y., Filion K.B., Azoulay L., Suissa S. (2018). Sulfonylureas as second line drugs in type 2 diabetes and the risk of cardiovascular and hypoglycaemic events: Population based cohort study. BMJ.

[95] Azoulay L., Suissa S. (2017). Sulfonylureas and the Risks of Cardiovascular Events and Death: A Methodological Meta-Regression Analysis of the Observational Studies. Diabetes Care.

[96] Buse J.B., Wexler D.J., Tsapas A., Rossing P., Mingrone G., Mathieu C., D’Alessio D.A., Davies M.J. (2020). 2019 Update to: Management of Hyperglycemia in Type 2 Diabetes, 2018. A Consensus Report by the American Diabetes Association (ADA) and the European Association for the Study of Diabetes (EASD). Diabetes Care.

[97] Chun J., Strong J., Urquhart S. (2019). Insulin Initiation and Titration in Patients with Type 2 Diabetes. Diabetes Spectr..

[98] Holmes R.S., Crabtree E., McDonagh M.S. (2019). Comparative effectiveness and harms of long-acting insulins for type 1 and type 2 diabetes: A systematic review and meta-analysis. Diabetes Obes. Metab..

[99] Wysham C., Bhargava A., Chaykin L., de la Rosa R., Handelsman Y., Troelsen L.N., Kvist K., Norwood P. (2017). Effect of Insulin Degludec vs Insulin Glargine U100 on Hypoglycemia in Patients with Type 2 Diabetes: The SWITCH 2 Randomized Clinical Trial. Jama.

[100] Vora J., Christensen T., Rana A., Bain S.C. (2014). Insulin degludec versus insulin glargine in type 1 and type 2 diabetes mellitus: A meta-analysis of endpoints in phase 3a trials. Diabetes Ther..

[101] Morris A. (2018). Closed-loop insulin delivery has wide-ranging benefits. Nat. Rev. Endocrinol..

[102] El-Khatib F.H., Balliro C., Hillard M.A., Magyar K.L., Ekhlaspour L., Sinha M., Mondesir D., Esmaeili A., Hartigan C., Thompson M.J. (2017). Home use of a bihormonal bionic pancreas versus insulin pump therapy in adults with type 1 diabetes: A multicentre randomised crossover trial. Lancet.

[103] Berget C., Messer L.H., Forlenza G.P. (2019). A Clinical Overview of Insulin Pump Therapy for the Management of Diabetes: Past, Present, and Future of Intensive Therapy. Diabetes Spectr..

[104] NICE Indicators for the NICE Menu for the QOF. https://www.nice.org.uk/Media/Default/Standards-and-indicators/QOF%20Indicator%20Key%20documents/NM141-diabetes-guidance.pdf.

[105] American Diabetes Association (2020). 6 Glycemic Targets: Standards of Medical Care in Diabetes—2021. Diabetes Care.

[106] Patel A., MacMahon S., Chalmers J., Neal B., Billot L., Woodward M., Marre M., Cooper M., Glasziou P., Grobbee D. (2008). Intensive blood glucose control and vascular outcomes in patients with type 2 diabetes. N. Engl. J. Med..

[107] Duckworth W., Abraira C., Moritz T., Reda D., Emanuele N., Reaven P.D., Zieve F.J., Marks J., Davis S.N., Hayward R. (2009). Glucose control and vascular complications in veterans with type 2 diabetes. N. Engl. J. Med..

[108] Chew E.Y., Ambrosius W.T., Davis M.D., Danis R.P., Gangaputra S., Greven C.M., Hubbard L., Esser B.A., Lovato J.F., Perdue L.H. (2010). Effects of medical therapies on retinopathy progression in type 2 diabetes. N. Engl. J. Med..

[109] Calles-Escandón J., Lovato L.C., Simons-Morton D.G., Kendall D.M., Pop-Busui R., Cohen R.M., Bonds D.E., Fonseca V.A., Ismail-Beigi F., Banerji M.A. (2010). Effect of intensive compared with standard glycemia treatment strategies on mortality by baseline subgroup characteristics: The Action to Control Cardiovascular Risk in Diabetes (ACCORD) trial. Diabetes Care.

